# First a Surgeon; Then a Son (Clinically)

**Published:** 1899-09

**Authors:** 


### FIRST A SURGEON; THEN A SON (CLINICALLY).

At a Chicago hospital, a surgeon, assisted by his son, also a surgeon, was stricken with heart disease while performing an operation. The attack came at the most critical moment of the operation, and any delay in completing it meant probable death for the patient, and very great peril as a matter of certainty. The son had to choose, and instantly, between devoting his attention and his skill to the unconscious form on the table, or to the other unconscious form on the floor—between a person to whom he owed a professional duty, and his own father. Presumably, the son knew of his father's malady and that the seizure was likely to be speedily fatal. The son did not hesitate. He picked up the fallen scalpel and completed the operation, allowing his father meanwhile to be removed and cared for by others. But, sad to relate, when the son's work was done and the patient safe, the father was dead.

The subjoined comment of a New York daily paper is a just tribute to the trying situation and decision of the son, and to the professional ideal of paramount duty, on which alone the trust of humanity can repose.

"It seems to us that the young surgeon deserves the highest commendation and that his act was truest heroism. Under great stress of feeling he reached without delay a sound conclusion and accepted it. There was nothing he could do for his father that others could not do, while only he knew just how the operation in progress had been planned, the minute details of the patient's condition, and the course indicated by previous study of the case as safest and most hopeful. Any other surgeon would inevitably have been delayed by the necessity of examining into the steps already taken, and so the patient's chance would have been decreased with no practical benefit to the original operator. Consciously or unconsciously, the son reasoned all this out, retained command of his nerves and feelings, deferred the manifestations of his filial anxiety—and did his duty."—*Modern Medical Science,* July, 1899.

